# Triple-negative and Her2-positive breast cancer in women aged 70 and over: prognostic impact of age according to treatment

**DOI:** 10.3389/fonc.2023.1287253

**Published:** 2023-12-15

**Authors:** Gilles Houvenaeghel, Monique Cohen, Anthony Gonçalves, Axel Berthelot, Marie Pierre Chauvet, Christelle Faure, Jean Marc Classe, Eva Jouve, Laura Sabiani, Marie Bannier, Louis Tassy, Marc Martino, Agnès Tallet, Alexandre de Nonneville

**Affiliations:** ^1^ Department of Surgical Oncology, Cancer Research Center of Marseille (CRCM), Institut Paoli−Calmettes, Aix-Marseille Univ, CNRS, INSERM, Marseille, France; ^2^ Department of Medical Oncology, Cancer Research Center of Marseille (CRCM), Institut Paoli−Calmettes, Aix-Marseille Univ, CNRS, INSERM, Marseille, France; ^3^ Surgical Oncology Department, Centre Oscar Lambret, Lille, France; ^4^ Surgical Oncology Department, Centre Léon Bérard, Lyon, France; ^5^ Institut René Gauducheau, Site hospitalier Nord, St Herblain, France; ^6^ Surgical Oncology Department, Centre Claudius Regaud, Toulouse, France; ^7^ Department of Radiotherapy, Cancer Research Center of Marseille (CRCM), Institut Paoli−Calmettes, Aix-Marseille Univ, CNRS, INSERM, Marseille, France

**Keywords:** breast cancer, triple negative, HER2-positive, elderly patients, geriatric oncology

## Abstract

**Background:**

Elderly breast cancer (BC) patients have been underrepresented in clinical trials whereas ~60% of deaths from BC occur in women aged 70 years and older. Only limited data are available on the prognostic impact of age according to treatment, especially in the triple-negative (TN) and Her2-positive because of the lower frequency of these subtypes in elderly patients. We report herein the results of a multicenter retrospective study analyzing the prognostic impact of age according to treatment delivered in TN and Her2-positive BC patients of 70 years or older, including comparison by age groups.

**Methods:**

The medical records of 31,473 patients treated from January 1991 to December 2018 were retrieved from 13 French cancer centers for retrospective analysis. Our study population included all ≥70 patients with TN or Her2-positive BC treated by upfront surgery. Three age categories were determined: 70-74, 75-80, and > 80 years.

**Results:**

Of 528 patients included, 243 patients were 70-74 years old (46%), 172 were 75-80 years (32.6%) and 113 were >80 years (21.4%). Half the population (51.9%, 274 patients) were TN, 30.1% (159) Her2-positive/hormone receptors (HR)-positive, and, 18% (95) Her2-positive/endocrine receptors (ER)-negative BC. Advanced tumor stage was associated with older age but no other prognostic factors (tumor subtype, tumor grade, LVI). Adjuvant chemotherapy delivery was inversely proportional to age. With 49 months median follow-up, all patient outcomes (overall survival (OS), disease-free survival (DFS), breast cancer-specific survival (BCSS), and recurrence-free survival (RFS)) significantly decreased as age increased. In multivariate analysis, age >80, pT2-3 sizes, axillary macrometastases, lymphovascular involvement, and HR-negativity tumor negatively affected DFS and OS. Comparison between age >80 and <=80 years old showed worse RFS in patients aged > 80 (HR=1.771, p=0.031).

**Conclusion:**

TN and Her2-positive subtypes occur at similar frequency in elderly patients. Older age is associated with more advanced tumor stage presentation. Chemotherapy use decreases with older age without worse other pejorative prognostic factors. Age >80, but not ≤80, independently affected DFS and OS.

## Highlights:

- TN and Her2-positive subtypes occur at a similar frequency in elderly patients
- Older age is associated with more advanced tumor stage presentation
- Chemotherapy use decreases with older age without worse other pejorative prognostic factors.
- Age >80, but not ≤80, independently affected DFS and OS.

## Introduction

Breast cancer (BC) incidence increases with age and more than one-third of patients with newly diagnosed breast cancer are aged 65 years or older (1). Although not fully consensual, a patient is considered elderly after the age of 70 according to several international recommendations (2). Between 1990 and 2023, the annual number of new BC in women doubled in France from 29,934 to 61,214 annual cases (+104%) (3). Half of this increase is attributable to population growth and aging (+26% and +21%, respectively). Age-specific trends show an average increase in breast cancer of approximately +1% per year for all ages, except for women in their 70s, for whom the increase is greater (+1.9%) (4). The decrease in breast cancer mortality is the result of major therapeutic advances (hormone therapy, taxanes, and anti-Her2 targeted therapy) associated with an increase in the proportion of cancers diagnosed at an early stage, notably through organized screening. However, this benefit in mortality appears to be less obvious in the elderly patient and 60% of deaths related to breast cancer occur in women aged 65 and over (4).

Elderly patients present more advanced BC, partly due to the absence of systemic screening (5, 6), display higher rates of hormone receptors (HR)-positive/Her2-negative cases than younger cohorts such as those included in pivotal studies (7), with higher triple-negative (TN) BC and Her2-positive tumors in young (<= 40 years old) and very young (<= 35 years old) patients (8). Elderly BC patients are often under-treated compared with younger BC patients (9–12) and present higher rates of recurrence and mortality (10, 13–17), with a 5-year survival rate of 82.4% in patients 70-79 and 74% in patients more than 80 (13, 17–19). To note that non-compliance to endocrine therapy and radiotherapy is higher in patients 80 years and older (16, 20–25). Most elderly patients are considered for upfront surgery, whatever the tumor phenotype and neo-adjuvant treatments seem less frequently offered to older patients, particularly those patients more than 80 years. However, an increasing proportion of elderly women appears as fit with few comorbidities and should be offered similar treatments to younger women. Hence, a significant proportion of older patients with TN and Her2-positive BC should receive neo-adjuvant chemotherapy (+/- Trastuzumab): cN1 or cN0 usN1 with positive axillary lymph node and cN0 pT2. Meanwhile, neo-adjuvant chemotherapy is discussed in patients with cN0 pT1c (mainly in 15-20 mm tumors) (26, 27). After up-front surgery, lymph node-positive or lymph node-negative and > pT1b patients should receive adjuvant chemotherapy in TN phenotypes (28, 29) and adjuvant chemotherapy and Trastuzumab in Her2-positive disease (28, 30).

There is a lack of data on TN and Her2-positive BC in elderly patients because of the lower frequency of these subtypes. We report herein the results of a multicenter retrospective study analyzing the prognostic impact of age according to treatment delivered in TN and Her2-positive BC patients of 70 years or older, including comparison by age groups (70-74, 75-80, and >80 years). Comparison between patients 70-80 years and >80-years, and results according to age groups for pT1, pN0 or pN0(i+) or pN1mi were analyzed.

## Methods

### Study design and data source

The medical records of 31,473 patients treated for invasive BC by up-front surgery were retrieved from the clinical databases of 13 cancer centers in France for retrospective analysis. All clinical variables analyzed in this study were retrieved from patient’s medical records. Up-front surgery was realized for 14,488 patients from January 1991 to December 2018, including 11,495 (79.3%) HR-positive Her2-negative BC, 1,232 (8.5%) TNBC, and 1,761 (12.1%) Her2-positive BC (614 HR-negative and 1,147 HR-positive). Patients ≥70 represented 19.2% (2,206/11,495), 22.2% (274/1,232), and 14.4% (254/1,761) of HR-positive Her2-negative, TN, and Her2-positive BC, respectively. Our study population included all ≥70 patients with TN or Her2-positive BC treated by upfront surgery (**Supplementary Figure 1**). Three categories of age were determined: 70-74, 75-80, and > 80 years.

Factors associated with adjuvant chemotherapy administration, type of surgery (breast conservative surgery or mastectomy, sentinel lymph node biopsy (SLNB) or axillary lymph node dissection (ALND)), radiation therapy delivery (regional nodal irradiation (RNI), post-mastectomy radiotherapy (PMRT)) were analyzed with univariate and multivariate analyses. Overall survival (OS), disease-free survival (DFS), and breast cancer-specific survival (BCSS) were assessed with univariate and multivariate analyses. In this large cohort of patients, chemotherapy regimens were not recorded. However, during this long period, chemotherapy differ, particularly before and since 2005. Consequently, we analyzed results also according these two periods.

### Pathological assessment

ER and Her2 status were determined according to French guidelines (immunohistochemistry (IHC) detection on formalin-fixed paraffin-embedded samples, of estrogen and/or progesterone receptors with a 10% threshold for ER positivity; Her2 positivity with a 3+ IHC score and/or Her2 amplification identified by *in situ* hybridization). Lymphovascular invasion (LVI), defined as tumor cells lying in an endothelium-lined space within the peritumoral area, were assessed by trained pathologists on examination of hematoxylin, eosin & safran (HES) slides (31).

### Statistical analysis

Overall survival (1), DFS (2), relapse-free survival (RFS, 3), and BCSS (4) were defined as the time interval from the date of surgery to (1) death or last follow-up, (2) any event (recurrence, metastasis, or death) or last follow-up, (3) local, regional, or distant recurrence whichever comes first or last follow-up, and (4) the date of cancer death or last follow-up, respectively. Patients lost to follow-up were considered alive at the date of last contact. The associations between categorical values were evaluated via χ^2^ tests. Factors significantly associated with pN status were determined by binary logistic regression adjusted for all significant variables determined by univariate analysis. Survival functions were calculated using the Kaplan-Meier method with differences assessed via the log-rank test. Multivariate survival analyses were performed using the Cox proportional-hazard-regression model adjusted for significant variables. Statistical significance was set at *p ≤* 0.05. Analyses were performed with SPSS-16.0 (SPSS-Inc., Chicago-Illinois, USA) and R version 3.2.4 software (http://www.cran.r-project.org/). All procedures performed in this study involving human participants were done by the French ethical standards and with the 2008 Helsinki Declaration. As this was a retrospective non-interventional study, no formal personal consent was required. Authorization to use the database was obtained from the strategic orientation committee of Paoli-Calmettes Institute (ClinicalTrials.gov NCT02869607).

## Results

### Patient characteristic

Five hundred twenty-eight patients fulfilled the inclusion criteria, including 243 patients 70-74 years old (46%), 172 75-80 years (32.6%), and 113 > 80 years (21.4%). Half the study population (51.9%, 274 patients) were TN, 30.1% (159) HR-positive and Her2-positive, and 18% (95) HR-negative and Her2-positive BC. The higher the age, the more the tumor stage was advanced (higher tumor size, more node involvement). Other prognostic factors (tumor subtype, tumor grade, LVI) were not significantly different between group ages (**Table 1**).

**Table 1:** Characteristics of patients in the three age groups.

Triple Negative & Her2+		Total		70-74		75-80		> 80		Chi 2
		Nb	%	Nb	%	Nb	%	Nb	%	p
All patients		528		243	46.0	172	32.6	113	21.4	
Subtype	ER- Her2-	274	51.9	131	53.9	78	45.3	65	57.5	0.222
	ER+ Her2+	159	30.1	72	29.6	60	34.9	27	23.9	
	ER- Her2+	95	18.0	40	16.5	34	19.8	21	18.6	
Breast surgery	Conservative	321	60.8	171	70.4	94	54.7	56	49.6	0.001
	Mastectomy	190	36.0	68	28.0	70	40.7	52	46.0	
	Unknown			4	1.6	8	4.7	5	4.4	
ALND	No	289	54.7	149	61.3	86	50.0	54	47.8	0.018
	Yes	239	45.3	94	38.7	86	50.0	59	52.2	
Radiotherapy	No	91	17.2	34	14.0	14.5	32	28.3		0.010
	Yes	409	77.5	196	80.7	80.8	74	65.5		
	unknown	28	5.3	13	5.3	4.7	7	6.2		
RNI	No	174	48.1	86	50.3	54	42.9	34	52.3	0.337
(n=362 known)	Yes	188	51.9	85	49.7	72	57.1	31	47.7	
Mastectomy	No RTH	67	35.3	25	36.8	19	27.1	23	44.2	0.141
	RTH	123	64.7	43	63.2	51	72.9	29	55.8	
AC	No	246	46.6	93	38.3	71	41.3	82	72.6	<0.0001
	Yes	282	53.4	150	61.7	101	58.7	31	27.4	
Endocrine therapy*	No	12	7.5	3	4.2	4	6.7	5	18.5	0.052
	Yes	147	92.5	69	95.8	56	93.3	22	81.5	
cT stage	T0	103	19.5	65	26.7	26	15.1	12	10.6	<0.0001
	T1	195	36.9	101	41.6	63	36.6	31	27.4	
	T2	168	31.8	56	23.0	63	36.6	49	43.4	
	T3	27	5.1	9	3.7	9	5.2	9	8.0	
	T4	5	0.9	0	0	2	1.2	3	2.7	
	Unknown	30	5.7	12	4.9	9	5.2	9	8.0	
pT	<= 20mm	273	51.7	154	63.4	76	44.2	43	38.1	<0.0001
	20-50 mm	211	40.0	73	30.0	83	48.3	55	48.7	
	> 50 mm	44	8.3	16	6.6	13	7.6	15	13.3	
pN	pN0	332	62.9	167	68.7	98	57.0	67	59.3	0.010
	pN0(i+)	10	1.9	5	2.1	4	2.3	1	0.9	
	pN1mi	36	6.8	17	7.0	16	9.3	3	2.7	
	pN1 macro	144	27.3	53	21.8	53	30.8	38	33.6	
	no axillary surgery	6	1.1	1	0.4	1	0.6	4	3.5	
Grade	1	42	8.0	26	10.7	11	6.4	5	4.4	0.065
	2	185	35.0	87	35.8	68	39.5	30	26.5	
	3	291	55.1	125	51.4	90	52.3	76	67.3	
	unknown	10	1.9	5	2.1	3	1.7	2	1.8	
LVI	No	360	68.2	167	68.7	114	66.3	79	69.9	0.145
	Yes	130	24.6	52	21.4	49	28.5	29	25.7	
	Unknown	38	7.2	24	9.9	9	5.2	5	4.4	
Local Recurrence	No	507	96.0	233	95.9	166	96.5	108	95.6	0.914
	Yes	21	4.0	10	4.1	6	3.5	5	4.4	
Metastases	No	457	86.6	213	87.7	153	89.0	91	80.5	0.099
	Yes	71	13.4	30	12.3	19	11.0	22	19.5	
Recurrence	No	437	82.8	204	84.0	146	84.9	87	77.0	0.181
	Yes	91	17.2	39	16.0	26	15.1	26	23.0	
Death	No	423	80.1	204	84.0	140	81.4	79	69.9	0.007
	Yes	105	19.9	39	16.0	32	18.6	34	30.1	
Periods	< 2005	138	26.1	76	31.3	39	22.7	23	20.4	0.042
	>= 2005	390	73.9	167	68.7	133	77.3	90	79.6	

AC, adjuvant chemotherapy; ALND, axillary lymph node dissection; cT stage, clinical T stage; ER, endocrine receptor; LVI, lymphovascular invasion; pN, pathologic nodal status; pT, pathologic tumor stage; RNI, regional nodal irradiation; RTH, radiotherapy.

*Endocrine therapy for ER+Her2+ patients.

### Treatments according to age groups

Adjuvant chemotherapy rates were inversely proportional to age (61.7%, 58.7%, and 27.4% in 70-74, 75-80, and > 80 years, respectively (p<0.0001)) (**Supplementary Table 1**). In binary logistic regression, adjuvant chemotherapy was less frequently performed in patients 75-80 years (OR=0.535, p=0.011) and >80 –years (OR=0.099, p<0.0001) (**Table 2**). Axillary node assessment (ALND or SLNB alone) was not significantly different according to age groups (**Table 3**). Although mastectomy appeared, at first glance, more frequently used in older patients (28%, 40.7%, and 46% in 70-74, 75-80, and > 80 years, respectively, p=0.001), it turned out not to be influenced by age after adjusting for confounding factors (**Tables 3**, **4**). Although post-mastectomy radiation therapy (PMRT) administration appeared independent of patients’ age, patients >80 years were less likely to receive regional nodal irradiation (RNI) (OR=0.281, p=0.001) (**Table 4**). Surprisingly, in 159 HR-positive Her2-positive BC, the endocrine therapy uses decreased with the increasing age group, with a lower rate in patients more than 80 years old (85.1% versus 93.3% and 95.8% in patients 70-74 years and 75-80 years, respectively) approaching statistical significance (p=0.052) (**Table 1**). Regarding periods of treatment, 138 patients were treated before 2005 (26.1%) and 390 since 2005. Tumor subtypes rates were 60.9% (84/138), 10.9% (15/138) and 28.3% (39/138) before 2005, 48.7% (190/390), 20.5% (80/390) and 30.8% (120/390) since 2005, for TNBC, ER- Her2+ and ER+ Her2+ BC, respectively (p=0.015).

**Table 2:** Adjuvant chemotherapy administration: multivariate analysis.

Adjuvant chemotherapy		p	OR	CI 95%	
				Inferior	Superior
**Grade**	Grade 1		1		
	Grade 2	0.034	2.443	1.068	5.591
	Grade 3	<0.0001	6.006	2.626	13.739
	unknown	0.516	0.465	0.046	4.686
**pN**	pN0		1		
	pN0(i+)	0.09	4.846	0.780	30.117
	pN1mi	0.001	5.647	2.005	15.906
	pN1macro	<0.0001	3.026	1.78	5.143
**Subtype**	TNBC		1		
	ER- Her2+	0.013	2.126	1.176	3.844
	ER+ Her2+	0.113	1.466	0.914	2.352
**pT**	pT1		1		
	pT2	0.008	1.885	1.179	3.013
** **	pT3-4	0.738	1.154	0.499	2.670
**LVI**	no LVI		1		
	LVI	0.298	1.324	0.780	2.248
	unknown	0.065	0.459	0.201	1.050
**Age**	70-74		1		
	75-80	0.011	0.535	0.331	0.865
** **	>80	<0.0001	0.099	0.054	0.184

LVI, lymphovascular invasion; pN, pathologic nodal status; pT, pathologic tumor stage; TNBC, triple negative breast cancer.

**Table 3:** Breast and axillary surgery: multivariate analysis.

Mastectomy vs BCS		p	OR	CI 95%	
				Inferior	Superior
**Grade**	Grade 1		1		
	Grade 2	0.017	4.105	1.288	13.084
	Grade 3	0.045	3.249	1.024	10.303
	unknown	0.209	3.705	0.480	28.590
**pN**	pN0		1		
	pN0(i+)	0.226	2.440	0.576	10.329
	pN1mi	0.369	1.474	0.632	3.439
	pN1macro	<0.0001	2.915	1.796	4.730
** **	no surgery	0.188	4.055	0.504	32.633
**Subtype**	TNBC		1		
	ER- Her2+	0.010	2.177	1.203	3.939
	ER+ Her2+	0.043	1.683	1.016	2.790
**cT stage**	cT0		1		
	1	0.334	0.731	0.387	1.380
	2	<0.0001	4.174	2.239	7.782
	3	<0.0001	12.568	3.674	42.994
** **	unknown	0.026	4.255	1.194	15.172
**Age**	70-74		1		
	75-80	0.352	1.267	0.769	2.088
	>80	0.337	1.326	0.746	2.356
**ALND**		p	OR	CI 95%	
** **				Inferior	Superior
**pN**	pN0		1		
	pN0(i+)	0.461	1.749	0.396	7.723
	pN1mi	<0.0001	7.491	3.280	17.110
	pN1macro	<0.0001	21.941	11.383	42.293
**Subtype**	TNBC		1		
	ER- Her2+	0.935	1.028	0.531	1.989
** **	ER+ Her2+	0.395	0.790	0.459	1.360
**cT**	cT0		1		
	1	<0.0001	3.605	1.768	7.349
	2	<0.0001	4.816	2.280	10.176
	3	0.010	7.766	1.644	36.678
	4	0.514	2.461	0.164	36.870
	unknown	0.005	8.246	1.874	36.286
**Surgery**	BCS		1		
	Mastectomy	<0.0001	2.910	1.701	4.978
** **	unknown	0.014	0.044	0.004	0.534
**Age**	70-74		1		
	75-80	0.636	1.138	0.666	1.943
** **	>80	0.479	1.261	0.663	2.398

ALND, axillary lymph node dissection; BCS, breast conservative surgery; cT stage, clinical T stage; pN, pathologic nodal status.

**Table 4:** PMRT and RNI: multivariate analysis.

PMRT		p	OR	CI 95%	
				Inferior	Superior
**pN**	pN0		1		
	pN0(i+)	0.532	1.854	0.267	12.868
	pN1mi	0.998	2,57E+09	0	.
	pN1macro	<0.0001	10.345	4.320	24.770
	no surgery	0.765	0.687	0.058	8.101
**pT**	pT1		1		
	pT2	0.018	3.009	1.209	7.485
** **	pT3-4	0.114	2.562	0.799	8.217
**LVI**	no LVI		1		
	LVI	0.596	1.288	0.504	3.292
	unknown	0.835	0.836	0.155	4.508
**Age**	70-74		1		
	75-80	0.941	0.966	0.385	2.422
** **	>80	0.078	0.423	0.162	1.103
Regional Nodal Irradiation		p	OR	CI 95%	
				Inferior	Superior
**pN**	pN0		1		
	pN0(i+)	0.089	3.599	0.823	15.748
	pN1mi	<0.0001	8.439	3.560	20.009
	pN1macro	<0.0001	19.218	10.211	36.172
**Subtype**	TNBC		1		
	ER- Her2+	0.707	1.134	0.588	2.186
** **	ER+ Her2+	0.802	0.927	0.515	1.670
**Surgery**	BCS		1		
	Mastectomy	0.920	1.030	0.578	1.834
	unknown	0.055	0.089	0.007	1.052
**pT**	pT1		1		
	pT2	0.130	1.531	0.882	2.657
** **	pT3-4	0.628	1.283	0.469	3.510
**LVI**	no LVI		1		
	LVI	0.238	1.418	0.794	2.533
	unknown	0.887	1.078	0.380	3.058
**Grade**	Grade 1		1		
	Grade 2	0.906	1.070	0.350	3.272
	Grade 3	0.226	1.943	0.664	5.687
** **	unknown	0.986	0.981	0.110	8.712
**Age**	70-74		1		
	75-80	0.932	1.024	0.585	1.794
** **	>80	0.001	0.281	0.135	0.583

BCS, breast conservative surgery; LVI, lymphovascular invasion; PMRT, post-mastectomy radiotherapy; pN, pathologic nodal status; pT, pathologic tumor stage; RNI, regional nodal irradiation; TNBC, triple negative breast cancer.

### Survival results, all patients

**Figure 1:**
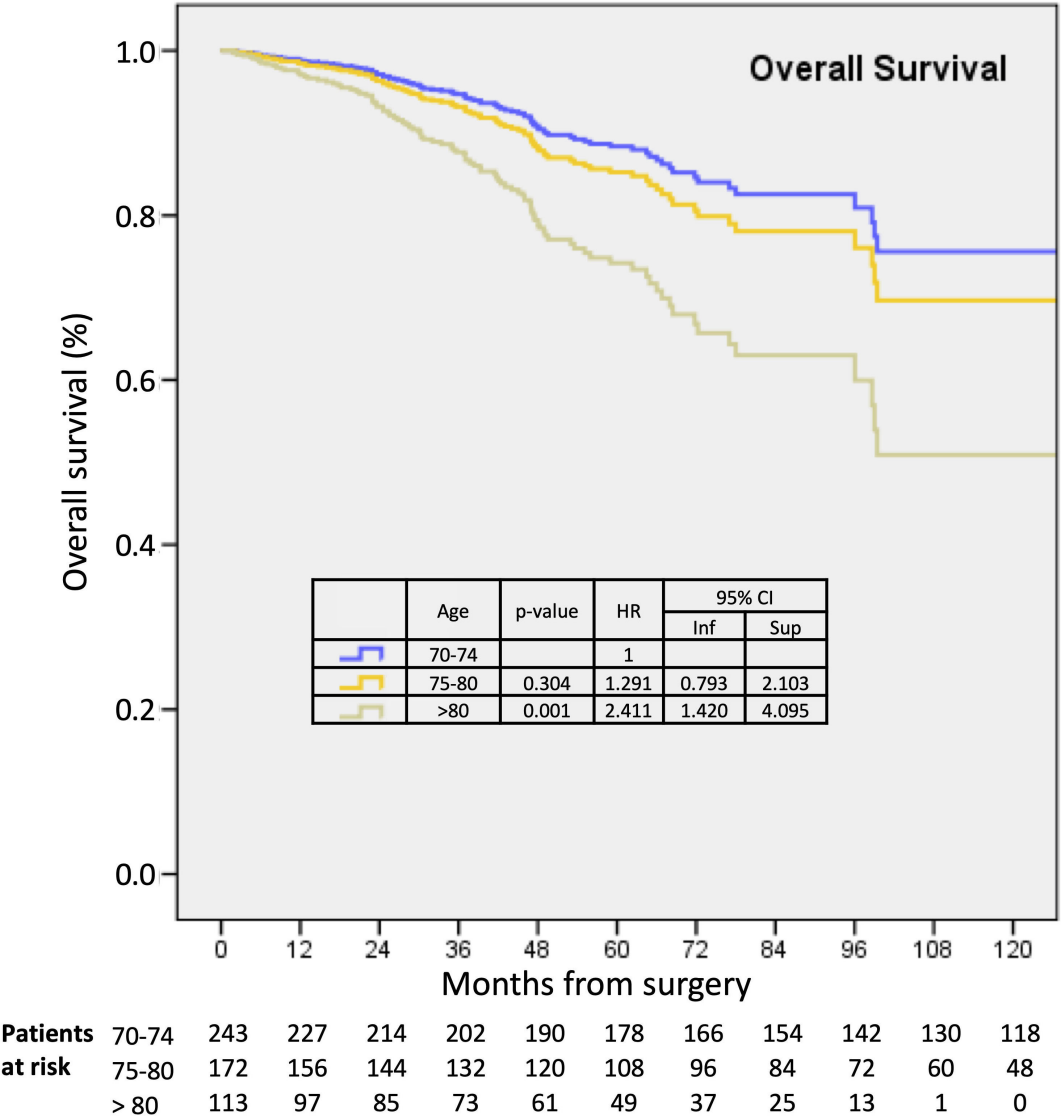
Kaplan-Meier survival estimates for overall survival in 70-74, 75-80, and >80 years old patients.

Median follow-up was 49 months (mean 51, 95% CI 48.3-53.7), with decreasing values ​​according to the three age groups (56.23, 49, 31.54). In univariate analysis, all patient outcomes (OS, DFS, BCSS, and RFS) significantly decreased as age increased, especially in patients older than 80 years (**Supplementary Table 2**). In multivariate analysis, age >80, pT2-3 sizes, axillary macrometastases, LVI, and HR-negative/Her2-positive tumor subtype, all negatively affected OS (**Figure 1**). Tumor size, axillary macrometastases, and LVI remained independent prognostic factors for DFS, BCSS, and RFS, whereas age > 80 did not. (**Table 5**). When periods were included in multivariate analysis, similar results were observed for patients over 80-years old for OS (HR=3.009, p<0.0001), DFS (HR=2.534, p<0.0001), RFS (HR=1.948, p=0.025) and BCSS (HR=2.255, p=0.030) and better results were observed for period ≥ 2005.

**Table 5:** OS, DFS and BCSS: multivariate analysis.

		Overall survival				Disease Free Survival				Breast Cancer Specific Survival				Recurrence Free Survival			
		p	HR	95% CI		p	HR	95% CI		p	HR	95% CI		p	HR	95% CI	
				Inf	Sup			Inf	Sup			Inf	Sup			Inf	Sup
**Age**	70-74		1								1				1		
	75-80	0.304	1.291	0.793	2.103	0.457	1.175	0.768	1.797	0.534	0.819	0.438	1.534	0.377	0.793	0.473	1.328
** **	>80	0.001	2.411	1.420	4.095	<0.0001	2.481	1.595	3.860	0.212	1.558	0.777	3.124	0.112	1.576	0.900	2.759
**pT**	pT1		1								1				1		
	pT2	<0.0001	3.951	2.357	6.621	<0.0001	2.382	1.567	3.622	<0.0001	3.653	1.766	7.558	0.003	2.232	1.318	3.781
	pT3-4	<0.0001	5.149	2.643	10.030	<0.0001	3.317	1.878	5.856	0.001	4.952	1.973	12.427	0.001	3.345	1.646	6.798
**LVI**	no LVI		1								1				1		
	LVI	<0.0001	2.272	1.432	3.605	<0.0001	2.148	1.452	3.178	0.002	2.445	1.376	4.343	<0.0001	2.496	1.566	3.978
** **	unknown	0.010	2.587	1.261	5.309	0.039	1.997	1.034	3.855	0.817	1.189	0.275	5.144	0.862	0.900	0.274	2.958
**pN**	pN0		1								1				1		
	pN0(i+)	0.563	0.550	0.073	4.167	0.714	0.766	0.184	3.185	0.739	1.419	0.182	11.088	0.527	1.601	0.373	6.883
	pN1mi	0.147	0.406	0.120	1.372	0.119	0.475	0.186	1.210	0.491	0.585	0.127	2.689	0.709	0.814	0.277	2.392
	pN1macro	0.084	1.536	0.944	2.499	0.034	1.564	1.034	2.364	0.001	3.019	1.527	5.969	0.001	2.531	1.495	4.284
	no surgery	<0.0001	6.985	2.678	18.221	<0.0001	5.399	2.178	13.381	0.005	9.262	1.956	43.848	0.127	3.164	0.721	13.878
**Chemotherapy**	Yes vs No	0.089	0.670	0.422	1.063					0.229	0.689	0.375	1.264	0,079	0.645	0.395	1.052
**Subtype**	TNBC		1														
	ER- Her2+	0.027	1.800	1.071	3.027												
** **	ER+ Her2+	0.080	1.722	0.937	3.165												

LVI, lymphovascular invasion; pN, pathologic nodal status; pT, pathologic tumor stage; TNBC, triple negative breast cancer.

### Comparison between patients 70-80years and 80 years

When aggregating patients in two groups (less or more than 80 years), the univariate analysis still showed older patients to have shorter BCSS (3-year BCSS: 87.7% vs. 88.6%, p=0.006), as well as lower RFS (3-year RFS: 80.5% vs. 88.6%, p<0.0001).

**Figure 2:**
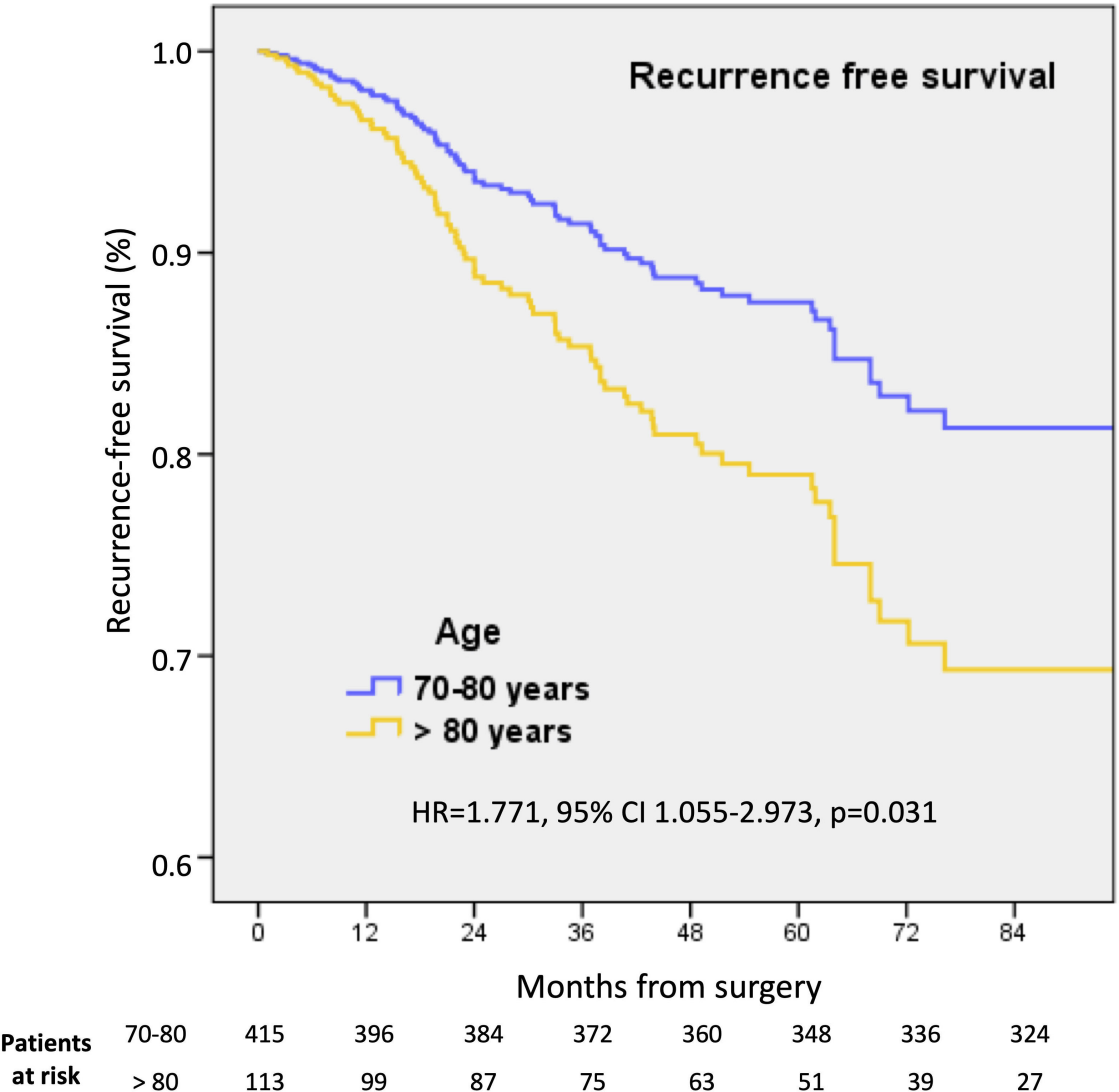
Kaplan-Meier survival estimates for recurrence-free survival in 70-80 and >80 years old patients.

However, after adjusting for tumor and patient characteristics (age, pT, pN, LVI, tumor subtype) and chemotherapy administration, we found no BCSS statistically significant difference in the two age groups (HR=1.633, 95% CI 0.855-3.121, p=0.138), but a worse RFS in patients aged > 80 (HR=1.771, 95% CI 1.055-2.973, p=0.031) (**Figure 2**). Usual prognostic factors still affected both BCSS and RFS, whereas chemotherapy did not. The tumor subtype impacted BCSS but not RFS (**Table 6**).

**Table 6:** BCSS and RFS: Comparison between patients 70-80 years and > 80-years in multivariate analysis.

		Recurrence Free Survival				Breast Cancer Specific Survival			
		p	HR	CI 95%		p	HR	CI 95%	
				Inf	Sup			Inf	Sup
**Age**	>80 vs 70-80	0.031	1.771	1.055	2.973	0.138	1.633	0.855	3.121
**pT**	pT1		1				1		
	pT2	0.002	2.254	1.338	3.798	<0.0001	3.705	1.792	7.663
** **	pT3	0.001	3.358	1.666	6.771	<0.0001	5.217	2.103	12.940
**pN**	pN0		1				1		
	pN0(i+)	0.404	1.870	0.429	8.147	0.698	1.508	0.189	12.012
	pN1mi	0.758	0.844	0.287	2.483	0.534	0.613	0.131	2.866
	pN1macro	<0.0001	2.601	1.545	4.380	0.001	3.077	1.564	6.057
	no surgery	0.186	2.726	0.616	12.066	0.010	7.722	1.616	36.892
**Chemotherapy**	No vs Yes	0.139	0.688	0.420	1.128	0.302	0.725	0.394	1.335
**LVI**	No		1				1		
	Yes	<0.0001	2.589	1.619	4.141	0.002	2.515	1.409	4.487
	unknown	0.898	0.925	0.281	3.049	0.742	1.281	0.294	5.593
**Subtype**	TNBC		1				1		
	ER- Her2+	0.100	0.599	0.325	1.102	0.361	0.737	0.382	1.419
** **	ER+ Her2+	0.156	0.696	0.422	1.148	0.025	0.453	0.226	0.905

BCSS, breast cancer specific survival; LVI, lymphovascular invasion; pN, pathologic nodal status; pT, pathologic tumor stage; RFS, recurrence free survival; TNBC, triple negative breast cancer.

### Patients with pT1, pN0 or pN0(i+) or pN1mi, TNBC or Her2-positive

We conducted the same analyses in the three age groups restricted to small tumors (pT1) with no or low nodal involvement (pN0-1mi) to find out whether de-escalation in adjuvant treatments can negatively impact elderly patients’ outcomes.

Two hundred thirty-four patients had pT1pN0-1mi TN (129), HR-Her2+ (30), or ER+Her2+ (75) BC, homogeneously represented in each age group (p=0.461), with 212 patients having pN0 disease (90.6%), 6 pN0(i+) (2.6%) and 16 pN1mi (6.8%) (**Supplementary Table 3**). In binary logistic regression, age >80 years was significantly associated with less adjuvant chemotherapy administration (OR=0.217, 95% CI 0.08-0.58, p=0.002). Adjuvant chemotherapy administration was significantly associated with pN1mi (OR=9.647, 2.01-46.22, p=0.005) and HR-negative Her2-positive subtype (OR=2.790, 1.12-6.94, p=0.027), without significant association with age 75-80 (OR=0.631, 0.32-1.23, p=0.179), pN0(i+), HR-positive Her2-positive BC and with LVI.

With a median follow-up was 55 months (mean 56.8, 95% CI 52.7-60.8), OS, DFS, and BCSS were not significantly different in the three age groups on multivariate analysis, whereas RFS tended to be lower in > 80 years patients (HR= 2.392, 95% CI: 0.893-6.410, p=0.083) (**Supplementary Figure 2**).

## Discussion

More than 50% of early-stage breast cancers are diagnosed in women aged more than 65 years, and >30% over the age of 70 years with these proportions in constant rise (32, 33). Elderly patients display higher rates of HR-positive/Her2-negative BC compared to TN and Her2-positive subtypes. Due to underrepresentation in clinical trials, only a few data dedicated to TN and Her2-positive elderly BC patients are available. We reported here histoclinical characteristics, treatments, and outcomes in 528 BC patients ≥70 years old including 274 with TN subtype and 254 with Her2-positive.

### Elderly patient characteristics

Consistently with available reports, our study found older patients having more advanced disease (but also more favorable prognostic factors, such as lower grade and LVI, and more frequently HR-positive tumors) (10, 13–17). We also found larger tumor size and lymph node involvement with increasing age. TN and Her2-positive were well balanced in elderly patients, being way less frequent than HR-positive/Her2-negative subtype, as reported in two large series. Plichta et al. (34) described a series of 156,240 ≤45 years patients and 210,095 ≥75 years patients, finding a TN rate of 14.9% vs. 8.2%, and a Her2-positive rate of 18.6% vs. 9.2%, respectively. Similarly, it was reported in a recent study including 235,368 early breast cancer patients, that the BC subtype distribution differs according to age, with an increase of Luminal BC and decrease of TNBC, Her-positive/HR-positive and Her2-positive/HR-negative BC, with higher age: 46.9%, 29.7%, 15.9%, and 7.5% respectively for age <30-years; 87%, 6.2%, 4.1% and 2.7% respectively for age 70-79 years; and 93.4%, 2.7%, 2.2% and 1.7% for patients age 80-years or older (35).

### Elderly patient management

Existing studies have reported elderly patients’ outcomes according to adjuvant treatment delivery, but only a few focused on TN and Her2-positive BC. Elderly patients are often undertreated due to multiple comorbidities as well as patients’ or relatives’ wishes. Jauhari et al. (36) recently reported as much as 5.6%, 11%, and 41.9% of surgery omission in 70-74, 75-79, and ≥ 80 years HR-positive BC patients, respectively, dropping to 3.8%, 3.7%, and 12.3% in HR-negative BC patients. Similar tendencies arose from the National Audit of Breast Cancer in Older Patients (NABCOP) (37) and National Health Service (NHS) (38). In a recent study of early breast cancer patients (35), surgery followed by chemotherapy was realized in 43.4% of all tumor subtypes, and neo-adjuvant chemotherapy in 30.9% (25.7% surgery without chemotherapy) for patients under 30 years; 21% and 2.9% (74.7%) and 1.6% of neo-adjuvant endocrine therapy for patients 70-79 years; 6.3% and 1.1% (87.2%) and 5.4% of neo-adjuvant endocrine therapy for patients 80-years or older. A common strategy in this patient population appears to rely on neo-adjuvant endocrine therapy in HR-positive in more or less debilitated patients, deemed with a short life expectancy (36, 39). Since we limited our study to patients referred for surgery, our data do not help in discussing this strategy. Nonetheless, contrary to several reports (16, 21, 22, 40–43), the type of surgery, including lymph node assessment, was independent of patients’ age in our study. This point is worth emphasizing since axillary surgery in clinically node-negative elderly patients remains moot. Indeed, axillary surgery, whether an ALND or even an SLNB, caters to some unneglectable adverse events (pain, numbness, loss of strength, decreased motion (44), which could substantially alter older patients’ quality of life. However, Corso G et al. (45) reported a matched analysis comparing axillary surgery to its omission in patients aged 70 years or older, stratified by age (70-74, 75-79, 80-84), with a significantly increased 10-y-risk of axillary lymph node recurrence, particularly in Luminal B, Her2-positive, and TNBC. Similarly, Marks et al. (46) showed that in an upfront surgery cohort of clinically node-positive elderly BC patients, the presence of less than 12 lymph nodes was associated with worse OS. Still, in a 1996 randomized trial (47), Avril et al. failed to show the safety of axillary assessment omission in early BC elderly patients. Nonetheless, several ongoing trials are currently evaluating SLNB omission in elderly patients with HR-positive BC. Although more frequent in higher-age patients in our univariate analysis, mastectomy was independent of age in multivariate analysis. Studies from a decade ago suggested that mastectomy prevails over breast-conserving surgery in elderly patients (9, 15, 16, 22, 25, 48), likely in the hope of avoiding adjuvant radiation therapy. Similarly, Jauhari et al. study (49) found the rate of mastectomy to increase with age (70–74, 75–79, and ≥ 80 years), irrespective of ER status. However, when comparing young (≤ 45 years) to elderly (≥ 75 years) patients, Plichta et al. (34) found the mastectomy rate to be higher in young patients (56% vs. 34%).

After breast-conserving surgery, adjuvant radiation therapy is standard of care since it improves the loco-regional recurrence rate and OS (EBCTCG 2014). Nonetheless, several studies reported on adjuvant radiation-therapy omission in the elderly due to fear of radiation toxicity and issues in transportation and mobility (16, 20, 21, 25, 48, 50). Several randomized studies in this setting have constantly found adjuvant radiation therapy to significantly improve local control without any impact on OS (51–57). On the other hand, a recent report by Tang et al. (58) showed that only 4.8% of elderly patients chose to decline adjuvant radiation therapy when asked for their preferred treatment option. PMRT still causes debates, even in young BC patients. Although the EBCTCG meta-analysis demonstrated its benefit in T1-2N1 BC patients who received chemotherapy (59), profuse literature data is currently challenging its actual need in intermediate-risk patients, irrespective of patient age. In our multicenter study, neither surgery nor adjuvant radiation therapy was altered by patient age, except RNI, which was less likely to be offered to older patients.

International guidelines recommend adjuvant chemotherapy (and Trastuzumab) in most patients with HR-negative or Her2-positive disease (60). In our study, we observed fewer adjuvant chemotherapy in patients 75-80 years (OR=0.533) and >80–years (OR=0.106). Few clinical trials have focused on elderly patients, and recommendations rely on the extrapolation of studies conducted in the general population, including a small proportion of elderly patients (61, 62). Multimorbidity competes with cancer on the outcome and the therapeutic ratio is narrower because they are at higher risk of side effects in this population. Recently, the results of the large phase III Unicancer ASTER 70s study have been reported (63). Brain and colleagues have shown that the addition of adjuvant chemotherapy to endocrine therapy does not result in a statistically significant overall survival (OS) benefit in patients older than 70 years with estrogen receptor (ER)-positive, Her2-negative breast cancer with a high tumor genomic grade index. However, in the CALGB 49907 study (64), adjuvant chemotherapy for patients over 65 years showed a beneficial impact, and the greatest benefit was reported in TNBC. In elderly patients, adjuvant treatment administration mainly depends on patient choice and associated comorbidities that may reduce tolerance and compliance with adjuvant treatments (19, 65–68). Moreover, toxicity prediction is challenging, and in a randomized trial, none of the multiple geriatric scores predicted tolerance of therapy (69). There is an increased likelihood of side effects, hospital admissions, and short-term mortality in elderly patients (70, 71).

### Elderly patient outcomes

OS and DFS were significantly associated with age > 80 years but without significant difference between age groups for BCSS. However, RFS was significantly lower for patients > 80 years in comparison with patients 70-80 years. For patients with pT1, pN0 or pN0(i+) or pN1mi BC, adjuvant chemotherapy administration was significantly associated and lower with age > 80 years (OR=0.217) but there was no significant OS, DFS, and BCSS difference between three age groups. We reported that systemic treatments were less likely to be administered to older patients. Multivariate analysis conducted on the whole population study found that age ≥ 80 did not affect BCSS or RFS, meaning that worse outcomes observed in this age population in the univariate analysis came from other prognostic factors, such as tumor size and lymph node involvements. Several studies suggest that adjuvant therapy may have a beneficial impact on patients with high-risk BC (node-positive or HR-negative) (39, 72–75). Higher BC recurrence rate (14, 48), higher distant recurrence rate (76), lower DFS and OS rates (16, 22), and higher disease-specific mortality (48) had been reported in elderly BC patients, whatever tumor subtypes. In 2002 patients ≥ 75 years matched by clinic-pathological and therapeutic factors, chemotherapy was associated with improved OS in Her2-positive BC but not in TNBC (77). In multivariate Cox survival analysis of 16,062 patients ≥ 70 years with resected TNBC, a beneficial impact of chemotherapy was observed for all groups of patients according to age subdivided into 5-year tranches and for TNBC with tumor size more than 20mm (78). Moreover, a propensity-matched analysis including 1,884 patients with TNBC, compared patients who received chemotherapy with those who were recommended to but did not receive chemotherapy and reported an OS improvement with chemotherapy (HR=0.69, 95% CI 0.60-0.80, p<0.0001). This benefit persisted after stratification for node-negative BC (HR=0.80, p=0.007), node-positive BC (HR=0.76, p=0.006), and those with a comorbidity score >0 (HR=0.74, p=0.013). In the same way, Tang et al. (79) have shown in elderly primary operable TNBC patients (≥70 years old) that age (HR=1.03 per year, p<0.001), T1c (HR=2.95) and chemotherapy (HR=0.79, p=0.035) were associated with lesser cancer-specific survival.

Although adjuvant trastuzumab is beneficial regardless of age, anti-Her2 adjuvant therapy remains only little evaluated in elderly patients (80, 81). The standard approach in elderly patients with early Her2-positive BC is one year of trastuzumab, combined with chemotherapy including docetaxel or weekly paclitaxel. However, the use of chemotherapy-free regimen can be proposed in frail patients (82), considering that age is associated with increased cardiac toxicity, particularly for patients aged 80 years or older with comorbidities (83, 84). Endocrine therapy is indicated in HR-positive BC, regardless of age, with aromatase inhibitors for 5 years, but could be omitted in patients with very low risk (85).

Our analysis in 234 pT1 pN0/1mi identified higher rates of chemotherapy in patients ≤80 years, with HR-negative Her2-positive, and with pN1mi tumors. OS, DFS, RFS, and OS were not significatively impacted by age groups. Only a few data have been reported regarding patients with small tumors without macroscopic lymph node involvements, and we noted in a previous study that tumor size may not be the main prognostic factor in T1 BC (86). While the need for trastuzumab-based adjuvant chemotherapy may be disputed in pT1a-b HR-positive/Her2-positive tumors (30, 87), chemotherapy is rarely omitted in node-negative TN BC larger than 5 mm. However, we recently failed to identify a significant advantage for adjuvant chemotherapy in pT1abN0 TNBC patients (29) and this systemic treatment might be discussed for elderly patients with tumors <1cm.

Our study has several limitations. Due to the retrospective design of the study, some valuable variables are missing from our analysis, including details of chemotherapy protocols on this 27-years’ time frame, collection of geriatric data, quality of life data, treatment acceptability, and socioeconomic data. Indeed, the study sample might be biased by the selection of patients eligible to surgery. The lack of data on patients comorbidities might be one of the principal limitations in this elderly population, because of the potential increase in the risk of chemotherapy-related complications, the effect on patients outcomes, as well as the influence that can occurs on treatment adaptations.

## Conclusion

TN and Her2-positive subtypes occur at a similar frequency in elderly patients. Older age is associated with more advanced tumor stage presentation. Chemotherapy use decreases with older age without being affected by other pejorative prognostic factors. This may reflect oncologists’ uncertainty when making management decisions in elderly patients, and the need to optimize BC management for these patients who may be fit, but where multimorbidity may also compete with cancer on outcomes.

## Data availability statement

The original contributions presented in the study are included in the article/**Supplementary Material**. Further inquiries can be directed to the corresponding author.

## Ethics statement

The studies involving humans were approved by strategic orientation committee of Paoli-Calmettes Institute. The studies were conducted in accordance with the local legislation and institutional requirements. Written informed consent for participation was not required from the participants or the participants’ legal guardians/next of kin in accordance with the national legislation and institutional requirements.

## Author contributions

GH: Conceptualization, Data curation, Formal analysis, Investigation, Methodology, Resources, Supervision, Validation, Writing – original draft, Writing – review & editing. MC: Resources, Writing – review & editing. AG: Resources, Writing – review & editing. AB: Resources, Writing – review & editing. MPC: Resources, Writing – review & editing. CF: Resources, Writing – review & editing. JC: Resources, Writing – review & editing. EJ: Resources, Writing – review & editing. LS: Resources, Writing – review & editing. MB: Resources, Writing – review & editing. LT: Resources, Writing – review & editing. MM: Resources, Writing – review & editing. AT: Resources, Writing – original draft, Writing – review & editing. AN: Conceptualization, Resources, Validation, Visualization, Writing – original draft, Writing – review & editing.

## Supplementary material

Supplementary Figure 1 Consort diagram showing patients selection.

Supplementary Figure 2 Kaplan-Meier survival estimates for recurrence-free survival in 70-80 and >80 years old patients with pT1 pN0, pN0(i+), and pN1mi tumors.

Supplementary Table 1 Factors associated with adjuvant chemotherapy in univariate analysis.

Supplementary Table 2 Overall Survival, Disease-Free Survival, Breast Cancer Specific Survival, and Recurrence Free Survival results in univariate analysis.

Supplementary Table 3 Characteristics of 234 patients pT1 pN0, pN0(i+), pN1mi and adjuvant chemotherapy administration.
