# Supplementary figures and images for: Triple-negative and Her2-positive breast cancer in women aged 70 and over: prognostic impact of age according to treatment
Source: Front Oncol. 2023 Dec 15;13:1287253. doi: 10.3389/fonc.2023.1287253 (PMC10757327; doi:10.3389/fonc.2023.1287253)

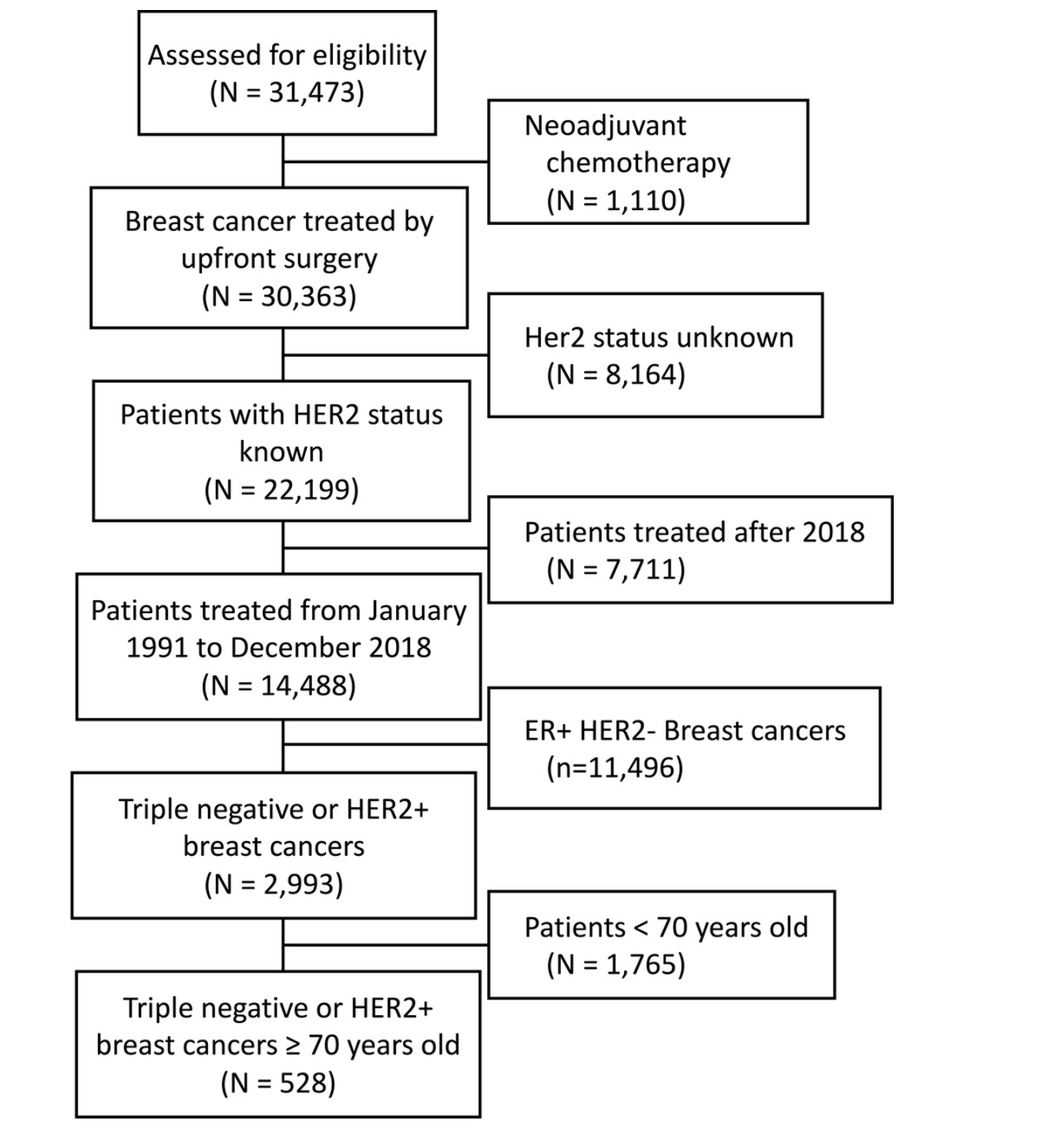

Supplement: Supplementary Figure 1 — Consort diagram showing patients selection. [file Image_1.tif]
