# Supplementary material for: Triple-negative and Her2-positive breast cancer in women aged 70 and over: prognostic impact of age according to treatment
Source: Front Oncol. 2023 Dec 15;13:1287253. doi: 10.3389/fonc.2023.1287253 (PMC10757327; doi:10.3389/fonc.2023.1287253)
Supplement: Supplementary Table 1 — Factors associated with adjuvant chemotherapy in univariate analysis. [file Table_1.docx]

Supplementary Table 1. Factors associated with adjuvant chemotherapy in univariate analysis.

| **Adjuvant Chemotherapy** | | **No** |  | **Yes** |  | **Chi 2** |
| --- | --- | --- | --- | --- | --- | --- |
|  |  | Nb | % | Nb | % | *p* |
| All patients |  | 246 | 46.6 | 282 | 53.4 |  |
| Breast surgery | Conservative | 163 | 66.3 | 158 | 56.0 | **<0.0001** |
|  | Mastectomy | 67 | 27.2 | 123 | 43.6 |  |
|  | Unknown | 16 | 6.5 | 1 | 5.9 |  |
| Age | 70-74 | 93 | 37.8 | 150 | 53.2 | **<0.0001** |
|  | 75-80 | 71 | 28.9 | 101 | 35.8 |  |
|  | > 80 | 82 | 33.3 | 31 | 11.0 |  |
| pN | pN0 | 191 | 77.6 | 141 | 50.0 | **<0.0001** |
|  | pN0(i+) | 2 | 0.8 | 8 | 2.8 |  |
|  | pN1mi | 5 | 2.0 | 31 | 11.0 |  |
|  | pN1 macro | 42 | 17.1 | 102 | 36.2 |  |
|  | no axillary surgery | 6 | 2.4 | 0 | 0 |  |
| cT stage | T0 | 51 | 20.7 | 52 | 18.4 | 0.006 |
|  | T1 | 93 | 37.8 | 102 | 36.2 |  |
|  | T2 | 64 | 26.0 | 104 | 36.9 |  |
|  | T3 | 13 | 5.3 | 14 | 5.0 |  |
|  | T4 | 2 | 0.8 | 3 | 1.1 |  |
|  | Unknown | 23 | 9.3 | 7 | 2.5 |  |
| LVI | No | 179 | 72.8 | 181 | 64.2 | **<0.0001** |
|  | Yes | 40 | 16.3 | 90 | 31.9 |  |
|  | Unknown | 27 | 11.0 | 11 | 3.9 |  |
| pT | pT1 | 152 | 61.8 | 121 | 42.9 | **<0.0001** |
|  | pT2 | 75 | 30.5 | 136 | 48.2 |  |
|  | pT3 | 19 | 7.7 | 25 | 8.9 |  |
| Grade | 1 | 32 | 13.0 | 10 | 3.5 | **<0.0001** |
|  | 2 | 99 | 40.2 | 86 | 30.5 |  |
|  | 3 | 106 | 43.1 | 185 | 65.6 |  |
|  | unknown | 9 | 3.7 | 1 | 0.4 |  |
| Subtype | TNBC | 150 | 61.0 | 124 | 44.0 | **<0.0001** |
|  | ER- Her2+ | 26 | 10.6 | 69 | 24.5 |  |
|  | ER+ Her2+ | 70 | 28.5 | 89 | 31.6 |  |

Legend: cT stage: clinical T stage, ER: endocrine receptor, LVI: lymphovascular invasion, pN: pathologic nodal status, pT: pathologic tumor stage, TNBC: triple negative breast cancer.

Supplementary Table 2. Patient’s outcomes in univariate analysis.

|  | **RFS** | | | **Disease Free Survival** | | | **Overall Survival** | | | **BCSS** | | |
| --- | --- | --- | --- | --- | --- | --- | --- | --- | --- | --- | --- | --- |
|  | % | SD | at-risk | % | SD | at-risk | % | SD | at-risk | % | SD | at-risk |
| **70-74 years** |  |  |  |  |  |  |  |  |  |  |  |  |
| 3-years | 88.3 | 2.2 | 172 | 87.3 | 2.2 | 172 | 93.5 | 1.7 | 184 | 94.5 | 1.5 | 172 |
| 5-years | 84.1 | 2.6 | 110 | 80.7 | 2.8 | 109 | 85.9 | 2.6 | 116 | 88.7 | 2.3 | 109 |
| 7-years | 80.0 | 3.2 | 44 | 73.7 | 3.7 | 44 | 79.7 | 3.4 | 49 | 73.7 | 2.9 | 49 |
| **75-80 years** |  |  |  |  |  |  |  |  |  |  |  |  |
| 3-years | 88.8 | 2.7 | 107 | 82.0 | 3.2 | 106 | 89.3 | 2.6 | 117 | 95.6 | 1.8 | 117 |
| 5-years | 85.2 | 3.1 | 62 | 75.7 | 3.7 | 61 | 77.4 | 3.8 | 65 | 85.9 | 3.3 | 65 |
| 7-years | 75.4 | 5.4 | 10 | 63.4 | 6.0 | 11 | 69.9 | 6.1 | 11 | 81.4 | 5.4 | 10 |
| **> 80 years** |  |  |  |  |  |  |  |  |  |  |  |  |
| 3-years | 80.5 | 4.3 | 47 | 69.4 | 4.9 | 48 | 76.5 | 4.5 | 53 | 87.7 | 3.7 | 52 |
| 5-years | 71.3 | 5.4 | 18 | 56.7 | 6.0 | 18 | 65.3 | 5.7 | 21 | 82.0 | 4.7 | 21 |
| 7-years | 51.4 | 11.5 | 2 | 37.5 | 9.4 | 3 | 50.9 | 8.6 | 4 | 69.2 | 9.3 | 3 |
| **Log-Rank** | 0.002 | | | <0.0001 | | | <0.0001 | | | 0.021 | | |

Legend: BCSS: breast cancer specific survival, RFS: recurrence free survival.

Supplementary Table 3. Characteristics of 234 patients pT1 pN0, pN0(i+), pN1mi and adjuvant chemotherapy administration.

| **Triple Negative & Her2+** | | Total |  | **A C** | | **No AC** | **Chi 2** |
| --- | --- | --- | --- | --- | --- | --- | --- |
|  |  | Nb | % | Nb | %* | Nb | p |
| All patients |  | 234 |  | 98 | 41.9 | 136 |  |
| Age groups | 70-74 | 137 | 58.5 | 66 | 48.2 | 71 | 0.008 |
|  | 75-80 | 61 | 26.1 | 25 | 41.0 | 36 |  |
|  | > 80 | 36 | 15.4 | 7 | 19.4 | 29 |  |
| Subtype | ER- Her2- | 129 | 55.1 | 47 | 36.4 | 82 | 0.010 |
|  | ER+ Her2+ | 75 | 32.1 | 31 | 41.3 | 10 |  |
|  | ER- Her2+ | 30 | 12.8 | 20 | 66.7 | 44 |  |
| Breast surgery | Conservative | 190 | 81.2 | 81 | 42.6 |  | 0.003 |
|  | Mastectomy | 31 | 13.2 | 17 | 54.8 |  |  |
|  | Unknown | 13 | 5.6 | 0 | 0 |  |  |
| ALND | No | 189 | 80.8 | 83 | 43.9 | 106 | 0.130 |
|  | Yes | 45 | 19.2 | 15 | 33.3 | 30 |  |
| Radiotherapy | No | 31 | 13.2 | 13 | 41.9 | 18 | 0.001 |
|  | Yes | 182 | 77.8 | 84 | 46.2 | 98 |  |
|  | unknown | 21 | 9.0 | 1 | 4.8 | 20 |  |
| RNI | No | 148 | 79.1 | 63 | 42.6 | 85 | 0.006 |
| (187 known) | Yes | 39 | 20.9 | 26 | 66.7 | 13 |  |
| Mastectomy | No RTH | 21 | 67.7 | 9 | 42.9 | 12 | 0.058 |
|  | RTH | 10 | 32.3 | 8 | 80.0 | 2 |  |
| Endocrine therapy | No | 5 | 6.7 | 2 | 40.0 | 3 | 0.664 |
|  | Yes | 70 | 93.3 | 29 | 41.4 | 41 |  |
| cT stage | T0 | 80 | 34.2 | 36 | 45.0 | 44 | 0.004 |
|  | T1 | 116 | 49.6 | 53 | 45.7 | 63 |  |
|  | T2 | 19 | 8.1 | 8 | 42.1 | 11 |  |
|  | T3 | 1 | 0.4 | 1 | 100 | 0 |  |
|  | Unknown | 18 | 7.7 | 0 | 0 | 18 |  |
| pN | pN0 | 212 | 90.6 | 79 | 37.3 |  | <0.0001 |
|  | pN0(i+) | 6 | 2.6 | 5 | 83.3 |  |  |
|  | pN1mi | 16 | 6.8 | 14 | 87.5 |  |  |
| Grade | 1 | 30 | 12.8 | 3 | 10.0 | 27 | <0.0001 |
|  | 2 | 100 | 42.7 | 36 | 36.0 | 64 |  |
|  | 3 | 99 | 42.3 | 59 | 59.6 | 40 |  |
|  | unknown | 5 | 2.1 | 0 | 0 | 5 |  |
| LVI | No | 177 | 75.6 | 75 | 42.4 | 102 | 0.006 |
|  | Yes | 30 | 12.8 | 18 | 60.0 | 12 |  |
|  | Unknown | 27 | 11.5 | 5 | 18.5 | 22 |  |
| Local Recurrence | Yes | 7 | 3.0 | 2 | 28.6 | 5 |  |
| Metastases | No | 221 | 94.4 | 94 | 42.5 | 127 | 0.297 |
|  | Yes | 13 | 5.6 | 4 | 30.8 | 9 |  |
| Recurrence | No | 210 | 89.7 | 90 | 42.9 | 120 | 0.251 |
|  | Yes | 24 | 10.3 | 8 | 33.3 | 16 |  |
| Death | No | 213 | 91.0 | 92 | 43.2 | 121 | 0.143 |
|  | Yes | 21 | 9.0 | 6 | 28.6 | 15 |  |
| Periods | < 2005 | 63 | 26.9 | 12 | 19.0 | 51 | <0.0001 |
|  | >= 2005 | 171 | 73.1 | 86 | 50.3 | 85 |  |

Legends: ER: endocrine receptor, ALND: axillary lymph node dissection, RNI: regional node irradiation, RTH: radiotherapy, AC: adjuvant chemotherapy, cT: clinical tumor stage, pN: pathologic nodal status, LVI: lympho-vascular invasion. * % AC.
